# The American and Colonial Journals: Pharmacy

**Published:** 1839-04

**Authors:** 


### PHARMACY.

### *Effervescent Magnesia.*

THIS saline aperient, commonly known in connexion with the name of its inventor, as "Moxon's Aperient Effervescent Magnesia," has enjoyed considerable reputation from its peculiar gratefulness to a fastidious stomach, as a remedy in indigestion, heartburn, nausea, &c. The manner here indicated is that by which a preparation very similar to the original article may be made. It is the imitation of Mr. E. Durand.

Take of Carbonate of magnesia, one part.

Sulphate of magnesia,

Bicarbonate of soda,

Tartrate of soda and potash,

Tartaric acid, } of each two parts.

These ingredients must be perfectly dried by expelling the water of crystallization, then reduced to powder, and finally mixed together. Inclose in dry bottles, with good corks adapted to them, and seal with wax. If there be the least moisture contained in the mixture, carbonic acid will be generated, and bursting of the bottles will follow. *Dose.*—A tea-spoonful in half a tumbler of water, drunk in a state of effervescence. *American Journal of Pharmacy. Jan.* 1838.

